# The effectiveness of a simple antimicrobial stewardship intervention in general practice in Australia: a pilot study

**DOI:** 10.1186/s12879-020-05309-8

**Published:** 2020-08-07

**Authors:** Alicia J. Neels, Aaron E. Bloch, Stella M. Gwini, Eugene Athan

**Affiliations:** 1 Department of Pharmacy, Barwon Health, PO BOX 281, Geelong, Victoria 3220 Australia; 2 Department of Infectious Disease, Barwon Health, Geelong, Victoria Australia; 3 Department of Research, Barwon Health, Geelong, Victoria Australia; 4 School of Medicine, Deakin University, Geelong, Australia

**Keywords:** Appropriate, Education, Antibiotic, Prescribing, Antimicrobial stewardship, General practice

## Abstract

**Background:**

Inappropriate and excessive antimicrobial prescribing can lead to antimicrobial resistance. Antimicrobial Stewardship (AMS) principles are not well established in general practice in Australia despite the relatively high rate of community antimicrobial prescribing. Few interventions have been implemented that have resulted in a significant reduction or improvement in antimicrobial prescribing by General Practitioners (GPs). This study was therefore conducted to assess the impact of a novel GP educational intervention on the appropriateness of antimicrobial prescriptions as well as GP compliance with antimicrobial prescription guidelines.

**Methods:**

In 2018, a simple GP educational intervention was rolled out in a large clinic with the aim of improving antimicrobial prescribing. It included face-to-face education sessions with GPs on AMS principles, antimicrobial resistance, current prescribing guidelines and microbiological testing. An antibiotic appropriateness audit on prescribing practice before and after the educational intervention was conducted. Data were summarised using percentages and compared across time points using Chi-squared tests and Poisson regression (results reported as risk ratios (RR) with 95% confidence intervals (CI)).

**Results:**

Data from 376 and 369 prescriptions in July 2016 and July 2018, respectively, were extracted. There were significant improvements in appropriate antimicrobial selection (73.9% vs 92.8%, RR = 1.26; 95% CI = 1.18–1.34), appropriate duration (53.1% vs 87.7%, RR = 1.65; 95% CI = 1.49–1.83) and compliance with guidelines (42.2% vs 58.5%, RR = 1.39, 95% CI = 1.19–1.61) post- intervention. Documentation of antimicrobial duration directions, patient follow-up as well as patient weight significantly increased after the intervention (*p* < 0.001). There was significant reduction in; prescriptions without a listed indication for antimicrobial therapy, prescriptions without appropriate accompanying microbiological tests and the provision of unnecessary repeat prescriptions (*p* < 0.001). Inappropriate antimicrobial prescriptions observed pre-intervention for medical termination of pregnancy ceased post-intervention.

**Conclusions:**

Auditing GP antimicrobial prescriptions identified prescribing practices inconsistent with Australian guidelines. However, implementation of a simple education program led to significantly improved antimicrobial prescribing by GPs. These findings indicate the important role of AMS and continued antimicrobial education within general practice.

## Background

Antimicrobial resistance is a global threat to human health [1]. Excessive and inappropriate antimicrobial prescribing is a major contributor to this problem [1]. Australia has a higher rate of antimicrobial prescribing compared to the average of countries in the Organisation for Economic Co-operation and Development [2]. In 2017, 41.5% of Australians were prescribed at least one antimicrobial through the Australian Pharmaceutical Benefits Scheme (PBS) [3] and many of these prescriptions were inconsistent with Australian prescribing guidelines [4]. General practitioners (GPs) provide the majority of primary healthcare in the community setting and write up to three quarters of annual antimicrobial prescriptions in the primary care setting [3, 4]. Despite this, national Antimicrobial Stewardship (AMS) programs have been mainly directed at hospitals and incorporated into accreditation standards to enforce monitoring of antimicrobial usage, appropriateness of prescribing and adherence to guidelines [5, 6]. In contrast, AMS is not a clinical requirement for general practice in Australia, however, the Royal Australian College of General Practitioners has committed to adopt antimicrobial resistance (AMR) education programs and AMS principles have been implemented in 2017 [7].

Recent research has explored factors that contribute to inappropriate antibiotic prescribing in general practice. These include automatic repeat prescriptions, inappropriate durations and quantities and the extended period of time during which a prescription may be filled (12 months after issuing) [5, 8–11]. Some prescriptions are dispensed more than 60 days after the prescription date [4], suggesting likely usage for an alternate indication to that intended by the prescriber [8, 9]. Perceived patient expectation has been identified as significantly influencing GPs to prescribe broad spectrum antimicrobials [12, 13], although this may be countered by interventions promoting shared decision-making with patients [13, 14]. Delayed prescription provision (where a prescription is provided only to be used in the event of clinical deterioration) is another useful collaborative tool [15–17] .

Interventions designed to improve antimicrobial prescribing have shown success amongst GP trainees and supervisors [18–22]. Additionally, international interventions focussing on factors influencing inappropriate prescribing have been shown to improve antibiotic prescribing over time [15, 23–26], particularly for respiratory tract infections [27–30]. These interventions include educational sessions [31, 32] and support material for GPs [33, 34]. In Australia, effective educational interventions have been multi-faceted, encompassing online modules [26, 35] and academic detailing (face to face education provided by qualified professionals) [10, 36].

We sought to establish a simple educational intervention for GPs using these evidence-based techniques, targeted towards bacterial resistance, relevant microbiological tests, empirical therapy and duration of therapy. This approach is recommended to improve antimicrobial prescribing among GPs [37]. This study sought to assess the effectiveness of this carefully designed educational intervention on baseline antibiotic prescribing habits in general practice, focusing on compliance with national guidelines and antibiotic appropriateness.

## Methods

### Design and study setting

An interventional study was conducted in a regional city in South West Victoria, Australia. Data were collected before and after an educational intervention. The intervention occurred in a general practice with an average of 15 GPs providing specialised services that included sexual health (including medical termination of pregnancy) and travel medicine.

### Data collection

All oral antibiotic prescriptions written at the GP clinic in July 2016 were identified using the GP prescribing software Best Practice®. A new audit tool was developed using REDcap (a web-based application for data entry) to record prescriber, patient, allergies, drug, repeats, directions, indication, free-text clinical assessment, microbiological and radiological requests and findings and to review appointment outcome details. Details of any clinical observations were recorded in the audit tool to assist with prescription analysis. Paediatric weight was recorded if documented within 3 months of the prescription generation. All data were collected retrospectively by the researchers.

A prescription assessment algorithm based on the Australian Therapeutic Guidelines: Antibiotic version 15 [38] was developed. The algorithm included checking the dosage, frequency, antimicrobial spectrum match, repeat prescription, and duplication of therapy against the stated indication. Cases were documented when antimicrobial therapy was given where the indication did not require such therapy.

Prescriptions were deemed compliant with guidelines when drug, dose, frequency and duration aligned with the national guidelines. Prescriptions were considered appropriate when the antimicrobial choice, dose and duration were a reasonable selection for the indication (according to the spectrum of activity, pharmacokinetics of the antimicrobial and the disease).

Where a duration of therapy was not specified on the prescription or in the consultation notes, the total quantity supplied was considered the therapeutic course duration. Number of repeat prescriptions permitted was recorded and ongoing therapy was recorded as long-term therapy. A repeat was deemed unnecessary if it provided a total duration of therapy that exceeded guideline recommendations.

Additionally, reviewers documented instances where microbiological and radiological tests were indicated. Prescriptions were excluded if no clinical notes were available to assist with the assessment, or if they were for residential care patients who did not physically attend the clinic for review.

The same data were collected following the intervention for prescriptions written in the month of July 2018 at the same practice.

### The intervention

Following the initial data collection, a one-hour face to face academic detailing session was provided to GPs in June 2018 where the results of the audit were presented. Additionally, education was provided regarding appropriate antibiotic prescribing for common presentations (in accordance with the Australian Therapeutic Guidelines: Antibiotic version 15 [38]), regional antibiogram and resistance patterns, relevant microbiological investigations and AMS techniques such as delayed prescribing. GPs were provided with individual hard copies of the Australian Therapeutic Guidelines: Antibiotic version 15 [38]. Allocation of a short-term expiry date for antimicrobial prescriptions was presented as a tactic to prevent inappropriate dispensing beyond the acute illness duration.

The intervention team comprised an Infectious Diseases physician (EA) and Antimicrobial Stewardship pharmacist (AN) who analysed the results of the baseline audit for compliance with guidelines, appropriateness of prescribing and common prescribing errors.

### Statistical analysis

Analysis was completed using Stata Statistical Software version 14® (StataCorp, 2015. College Station, TX: StataCorp LP) and descriptive statistics were applied to guideline compliance, appropriateness of therapy and other variables measured. Categorical data were summarised using frequencies and percentages whilst continuous/interval data were summarised using means and standard deviations.

A chi-square test was used to compare proportion of females, number of patients aged < 12 years and number of patients aged ≥65 years in the two cohorts. Guideline compliance and appropriateness were compared between the two groups using Poisson regression with robust sandwich error estimates and results were reported as risk ratios (RR) with 95% confidence intervals (CI).

## Results

### GP participation

The number of GPs who prescribed at least one antimicrobial prescription in 2016 and 2018 were 17 and 18 respectively. All staff were aware of the project and all clinic GPs were invited to attend the education intervention which occurred twice to maximise participation. Twelve GPs attended the education session; 8 of whom worked during both study periods. Two GPs who had worked across both periods were unable to attend. An additional 4 GPs worked only during the post-intervention audit period.

### Patient and prescription demographics

Three hundred eighty-seven patients were prescribed at least one antimicrobial in the pre-intervention period, compared to 386 patients in the post-intervention period. The number of prescriptions reviewed was 395 and 373 respectively. The number of antibiotic prescriptions as a proportion of all prescriptions prepared in 2016 was 5.0% and in 2018 was 4.0%. The patient demographics are presented in Table 1. There were no statistically significant differences in age or gender distributions between the two cohorts.

**Table 1:** Patient and prescription demographics

	July 2016	July 2018
**Patients prescribed antibiotics**	387	386
**Excluded patients prescribed antibiotics**	11	17
**Included patients prescribed antibiotics**	376	369
**Average patient age prescribed antibiotics (years)**	42.7 (SD = 25.7)	43.1 (SD = 25.2)
**Median patient age prescribed antibiotics (years) (IQR)**	41 (22, 65)	42 (24, 65)
**Female patients prescribed antibiotics (%)**	233 (62%)	252 (68%)
**Age < 12 years prescribed antibiotics (%)**	55 (14.6%)	47 (12.7%)
**Age ≥ 65 years prescribed antibiotics (%)**	104 (27.7%)	94 (25.5%)
**Total antibiotic prescriptions reviewed**	395	373
**1 antibiotic prescription**^a^	358	365
**2 antibiotic prescriptions**^a^	17	4
**3 antibiotic prescriptions**^a^	1	0
**Total GP attendances**	3573	4014
**Total prescriptions prepared (all drugs)**	7942	9279
**Included antibiotic prescriptions (percentage of all prescriptions)**	5.0%	4.0%
**GPs who prescribed ≥ 1 antibiotic**	19	18

^a^Number of antibiotic prescriptions provided to patients within the study period

2 antibiotic prescriptions = 2 different antibiotics prescribed

3 antibiotic prescriptions = 3 different antibiotics prescribed

*SD* standard deviation, *IQR* interquartile range (25th percentile, 75th percentile)

### Compliance with guidelines, appropriateness of drug choice and duration of therapy

Overall prescription compliance with guidelines increased from 42.2 to 58.5% (*p* < 0.001) following the intervention. Appropriate antibiotic selection improved from 73.9 to 92.8% (*p* < 0.001). Appropriate duration of therapy increased from 53.1 to 87.8% (*p* < 0.001) (Table 2).

**Table 2:** Prescription compliance with guidelines and appropriateness Pre vs Post intervention for all ages, patients < 65 years and patients > 65 years

All ages			
	Pre	Post	Change
	n/N (%)	n/N (%)	RR (95% CI) [*p*-values]
**Compliance**	160/379 (42.2)	185/316 (58.5)	1.39 (1.19–1.61) [*p* < 0.001]
**Appropriateness**			
Drug	291/394 (73.9)	336/362 (92.8)	1.26 (1.18–1.34) [*p* < 0.001]
Duration	205/386 (53.1)	316/360 (87.7)	1.65 (1.49–1.83) [*p* < 0.001]

Appropriateness of prescribing by individual antimicrobial is shown in Table 3. Significant improvements occurred for amoxicillin, doxycycline, cefalexin, metronidazole and azithromycin. Compliance with guidelines also improved broadly (Table 3).

**Table 3:** Antibiotic frequencies, appropriateness and compliance

	(A) Number of prescriptions			(B) Appropriateness of prescription^a^ ([B/A (assessable prescriptions)]%)			(C) Guideline compliance^a^ ([C/A (assessable prescriptions)]%)		
Antimicrobial	PRE n(%)	POST n(%)	***p*** value^**†**^	PRE n(%)	POST n(%)	***p*** value^**†**^	PRE n(%)	POST n(%)	***p*** value^**†**^
Amoxicillin	73 (18.5)	71 (19.0)	0.859	63 (86.3)	68 (98.5)	0.007	40 (56.3)	46 (76.6)	0.015
Doxycycline	71 (18.0)	66 (17.7)	0.942	62 (87.3)	63 (98.4)	0.014	40 (57.1)	52 (83.9)	0.001
Cefalexin	49 (12.4)	57 (15.3)	0.244	29 (59.2)	53 (98.1)	< 0.001	16 (34.0)	16 (31.4)	0.784
Amoxicillin/clavulanic acid	35 (8.9)	49 (13.1)	0.062	24 (68.6)	34 (72.3)	0.716	12 (36.4)	15 (35.7)	0.949
Trimethoprim	30 (7.6)	26 (7.0)	0.709	28 (93.3)	26 (100)	0.179	9 (31.0)	14 (60.9)	0.031
Roxithromycin	21 (5.3)	4 (1.1)	0.001	9 (42.9)	2 (50.0)	0.793	0 (0.0)	1 (33.3)	0.007
Erythromycin	15 (3.8)	7 (1.9)	0.115	9 (60.0)	5 (71.4)	0.605	1 (7.1)	0 (0.0)	0.004
Phenoxymethylpenicillin	14 (3.5)	8 (2.1)	0.241	13 (92.7)	6 (75.0)	0.246	11 (84.6)	2 (33.3)	0.025
Metronidazole	13 (3.3)	27 (7.2)	0.015	6 (46.2)	26 (96.3)	0.001	1 (9.1)	6 (18.7)	0.509
Cefaclor	12 (3.0)	6 (1.6)	0.198	2 (16.7)	3 (60.0)	0.074	1 (8.3)	0 (0.0)	0.468
Flucloxacillin	12 (3.0)	10 (2.7)	0.803	11 (91.7)	10 (100.0)	0.351	8 (72.7)	7 (70.0)	0.891
Azithromycin	11 (2.8)	4 (1.1)	0.091	4 (36.4)	4 (100.0)	0.029	3 (27.3)	2 (50.0)	0.410

Other (39 prescriptions, 38 prescriptions): norfloxacin, tinidazole, nitrofurantoin, ciprofloxacin, clindamycin, cefuroxime, fluconazole, ivermectin, minocycline, methenamine hippurate, clarithromycin, trimethoprim/sulfamethoxazole, dicloxacillin

^a^Some missing data ranging from 1 to 15 prescriptions. Percentages calculated based on assessable prescriptions

^†^*p* value for comparison of proportions between cohorts

Significant improvement occurred in the post-intervention group for respiratory tract infection prescribing for appropriateness of drug, duration and guideline compliance. Appropriate drug selection also improved significantly for ear, nose & throat, skin and soft tissue, urinary tract and medical prophylaxis indications such as malaria prophylaxis (Table 4). Guideline compliance and appropriate duration also improved significantly for a number of indications.

**Table 4:** Indication frequency by appropriateness of drug and guideline compliance

(A) Indication	(B) Cohort	(C) Number of prescriptions (% of total prescriptions)	(D) Appropriate drug ([D/C]%)^a^	(E) Guideline compliance ([E/C]%)^a^	(F) Appropriate duration ([F/C]%)^a^
**Respiratory**	**Pre**	**113 (28.6)**	**79 (69.9)**	**44 (38.9)**	**66 (58.4)**
	**post**	**95 (25.5)**	**80 (87.0)**	**59 (67.8)**	**87 (94.6)**
	**p value**^†^	**0.617**	**0.009**	**0.004**	**< 0.001**
**Ear, nose, throat**	**Pre**	**77 (19.5)**	**61 (81.3)**	**32 (43.8)**	**33 (44.0)**
	**post**	**76 (20.4)**	**71 (94.7)**	**33 (49.3)**	**67 (88.2)**
	**p value**^†^	**0.889**	**0.016**	**0.657**	**< 0.001**
**Skin and soft tissue**	**Pre**	**78 (19.7)**	**60 (76.9)**	**42 (56.8)**	**66 (84.6)**
	**post**	**69 (18.5)**	**63 (91.3)**	**35 (54.7)**	**63 (91.3)**
	**p value**^†^	**0.854**	**0.028**	**0.853**	**0.244**
**Urinary tract infection**	**Pre**	**53 (13.4)**	**47 (88.7)**	**16 (31.4)**	**17 (32.1)**
	**post**	**54 (14.5)**	**54 (100.0)**	**21 (44.7)**	**39 (72.2)**
	**p value**^†^	**0.870**	**0.011**	**0.411**	**0.005**
**Medical prophylaxis**	**Pre**	**39 (9.9)**	**18 (46.2)**	**9 (25.0)**	**4 (12.9)**
	**post**	**25 (6.7)**	**20 (95.2)**	**16 (84.2)**	**16 (88.9)**
	**p value**^†^	**0.657**	**0.001**	**0.003**	**0.002**
**Genital and sexually transmitted infections**	**Pre**	**20 (5.1)**	**16 (80.0)**	**8 (50.0)**	**11 (55.0)**
	**post**	**28 (7.5)**	**28 (100.0)**	**8 (72.7)**	**24 (85.7)**
	**p value**^†^	**0.739**	**0.014**	**0.351**	**0.048**
**Gastrointestinal**	**Pre**	**9 (2.3)**	**8 (88.9)**	**8 (88.9)**	**7 (77.8)**
	**post**	**20 (5.4)**	**17 (89.5)**	**10 (58.8)**	**16 (84.2)**
	**p value**^†^	**0.708**	**0.964**	**0.157**	**0.712**

^a^ Some missing data ranging from 1 to 17 prescriptions. Percentages calculated based on assessable prescriptions

Other indications: 7 prescriptions (2016), 6 prescriptions (2018)

^†^*p* value for comparison of proportions between cohorts

### Antimicrobials

Table 3 shows the prescription frequency of individual antibiotic drugs. Amoxicillin was the most frequently prescribed antibiotic, accounting for one-fifth of prescriptions during both periods, followed by doxycycline (18.0 and 17.7%), cefalexin (12.4 and 15.3%) and amoxicillin/clavulanic acid (8.9 and 13.1%). A significant reduction in the number of prescriptions for roxithromycin and an increase for metronidazole occurred between the two periods. Appropriateness and compliance of each antimicrobial are presented in Table 3.

### Indication for therapy

Table 4 details the indications for antimicrobial prescriptions by body system. The most frequent indications by body site of infection (pre, post) were respiratory (28.6, 25.5%), ear, nose & throat (19.5, 20.4%), skin and soft tissue (19.7, 18.5%) and urinary tract (13.4, 14.5%). There were 14 prescriptions for medical prophylaxis for medical termination of pregnancy (MTOP) in 2016 and none post intervention. Prescriptions generated for indications that do not require antimicrobial therapy reduced significantly post intervention (5.3 to 0.8%, *p* < 0.001). A documented indication for the antibiotic prescription was available in all but 3 prescriptions for both periods.

### Investigations

There was no significant difference in the number of microbiology requests printed (*n* = 88 for 2016, *n* = 101 for 2018, *p* = 0.123), however prescriptions where microbiological testing was required but not performed reduced from 116 (29.4%) to 30 prescriptions (8.0%) (*p* < 0.001).

The difference in the proportion of cases in which radiology was indicated but not performed was not significant (3.5% in 2016 and 5.4% in 2018).

### Repeat prescriptions

There was no significant difference in the total number of repeat prescriptions provided in 2016 and 2018 (88 and 101 respectively; *p* = 0.221). However, the number of prescriptions with unnecessary repeats provided was reduced significantly from 46 (11.6%) to 12 prescriptions (3.2%) (*p* < 0.001).

### Delayed prescribing and expiry dates

The use of delayed prescribing increased significantly following intervention, from 6 to 27 delayed prescriptions (*p* < 0.001). The inclusion of a GP-allocated prescription expiry date was not present in 2016 but occurred in 3 prescriptions in 2018 (*p* = 0.075).

### Paediatric prescriptions

The number of patients aged less than 12 years in 2016 and 2018 was similar (Table 1). Documentation of patient weight within 3 months of consultation improved from 29 (52.7%) 2016, to 34 (72.3%) in 2018 (*p* = 0.042). A weight was documented at the time of prescription in 6 (10.9%) cases in 2016 and 15 (31.9%) in 2018 (*p* = 0.009).

### Patient review

Documentation of a required follow up review during the initial antimicrobial prescription consultation increased significantly between 2016 and 2018; from 83 (21.0%) to 156 (41.8%) respectively, *p* < 0.001). A total of 107 patient follow up reviews occurred in 2016 compared with 117 in 2018.

### Prescribing full pack sizes and duration directions

Where a full pack was prescribed, documentation of duration either on the prescription itself, or in the consultation notes improved between 2016 and 2018 from 16.5 to 40.2% (*p* < 0.001). Provision of an exact number of required doses increased by 2% between the two time points (5.3 and 7.2% respectively, *p* = 0.276).

## Discussion

This study measured baseline GP antimicrobial prescribing habits; identifying common errors and areas for improvement. This information was subsequently used to provide feedback to GPs as part of an educational intervention designed to improve antimicrobial prescribing. The success of the intervention was demonstrated by significant improvement across all major outcome measures including: concordance with guidelines, appropriate antibiotic selection and course duration.

Analysis of baseline prescribing data and discussion with GPs in the educational session revealed some key issues. Some prescriptions followed superseded guidelines or manufacturers product information, indicating lack of awareness of current guideline recommendations [39]. GPs acknowledged their infrequent referral to current guidelines. In this study, GPs experienced restricted access to an electronic guideline as the clinic had only one shared account. Where clinical guidelines are electronically available, individual access is an important consideration. Utilisation of outdated guidelines may be more widespread as evidenced by the fact that roxithromycin and cefaclor are among the top 10 PBS-dispensed antibiotics in Australia, despite having become largely superseded in current Therapeutic guidelines [3, 4]. To remedy this issue, GPs were provided with individual hard copies of the Therapeutic Guidelines which was well received by the participants due to the difficulty accessing the clinic’s online version. Following the intervention there was a substantial reduction in roxithromycin and cefaclor prescriptions and the use of prescribing guidelines was documented in clinical notes. More broadly, antimicrobial prescribing in primary care would benefit from improved access to electronic guidelines (where available) and linking prescribing software to guideline recommendations.

Prescriptions are deemed compliant with guidelines when drug, dose, frequency and duration are correct. Whilst there was improvement in this measure post intervention, the overall rate of prescriptions compliant with guidelines remained relatively low at 59%, mirroring that observed in Australian hospitals (54% in 2017) [40]. Appropriate antibiotic selection also occurs more often than complete prescription compliance in Australian hospitals (73% in 2017) [40]. Ongoing auditing and education may improve GP compliance with guidelines. Further research to review the effectiveness of integrating guidelines into prescription software would be beneficial.

Following the intervention there was improvement in appropriate antibiotic duration with better documentation on the prescription and in the clinical notes. One such example was trimethoprim for cystitis in non-pregnant women, which is often prescribed as a full pack, with 7 days of therapy, rather than the guideline recommended 3 days [38]. Following intervention, guideline compliance for trimethoprim almost doubled. This highlights a broader challenge for AMS in primary healthcare as Australian antibiotic pack sizes rarely align with recommended treatment protocols. Without clear duration directions, patients may consume excess doses which may contribute to adverse effects or antimicrobial resistance [11]. Prescribing the exact duration of therapy is ideal and is possible for most solid preparations, regardless of the number of doses in the original manufacturers’ pack. A perceived reluctance to break packs by pharmacies is commonly theorised, though not substantiated [10, 11]. However, this strategy requires further consideration to ensure patients are not financially burdened by this practice due to discordance with the Australian PBS.

GPs occasionally used delayed prescribing tactics; most often through the provision of a prescription with advice to delay therapy for a specified duration, until microbiology results are communicated or if deterioration occurs. The increased use of these tactics post intervention demonstrates GP acceptance of this method to curb unnecessary antimicrobial usage. The study did not ascertain the outcomes of delayed prescribing on the percentage of delayed scripts that were dispensed, though previous work has demonstrated overall reduced antibiotic use [16].

Repeat prescriptions are ordered with most antibiotic prescriptions in Australia [4] and often provide a duration of therapy that exceeds guidelines [38]. Furthermore, patients often don’t receive clear instructions about when to fill a repeat prescription [8] and may inappropriately extend their antimicrobial [9]. GP prescribing software may contribute to unnecessary repeat provision with the default setting providing a repeat prescription [3, 9, 10]. The significant reduction of inappropriate repeat prescriptions in this study indicates the success of this antimicrobial stewardship intervention. Additionally, up to 20% of antibiotic repeats are dispensed several months after the original prescription date [3, 4]. To address this issue, one GP issued three prescriptions with a documented arbitrary expiry date. Though not recognised legally, this strategy may be effective in preventing the dispensing of antimicrobial prescriptions beyond the immediate acute illness and may be worth considering in future AMS interventions. Widespread uptake would require legislative change. Limiting antimicrobial courses to their appropriate duration is a pivotal aspect of AMS programs to limit adverse effects for patients and avoid the development of resistance [6, 41]. In 2020, access to repeats for common antimicrobials was restricted on the PBS to reduce inappropriate prescribing [42].

### Strengths and limitations

This study reviewed direct prescriber documentation and recorded indication, observations and patient communication for each prescription. This is in contrast to recent studies, which utilised antimicrobial dispensing on the PBS [25] or reports generated from prescribing systems [39, 43]. Clinical notes assisted reviewers in the assessment of each individual prescription. The detailed information gathered is a clear strength, though the time required for each assessment is a limitation as it provides a possible barrier to reproducing this study on a broader scale.

Provision of a prescription does not guarantee dispensing, so it is assumed that patients adhered to the treatment prescribed. The frequency of consultations that occurred due to an infection, but where no antimicrobial was prescribed, was not identified. However, it was observed that some prescription-generating consultations were preceded by a consultation where antibiotics were discussed and not supplied by the GP. Future studies are required to investigate the frequency of prescription denial by GPs, including where patients have requested an unnecessary prescription. This study relied on GP documentation to assess whether or not information was provided to the patient regarding their prescription or medical management. Verbal counselling that may have been provided to patients was not captured.

Changes in prescription appropriateness and adherence to guidelines by individual prescribers was not assessed in this study, but rather, the focus was on the impact of the intervention more generally across the practice. GP staff also changed between 2016 and 2018 and not all GPs attended the intervention (due to availability). However, this study intended to review overall prescribing at the clinic level and not by individual GPs.

Other AMS campaigns targeting prescribers were active during the study period which may contribute to the study results. Antibiotics Awareness Week is recognised annually to highlight the importance of appropriate prescribing to reduce the threat of AMR [44]. Additionally, the first Antimicrobial Use and Resistance in Australia (AURA) report was published in 2016 [44]. All clinic GPs were also aware that their antimicrobial prescribing would be audited post-intervention.

This pilot intervention study was conducted in a single large GP practice using an audit tool that was not validated. Study numbers were small and replication in additional practices will establish the generalisability of these findings. Long term sustainability of improved prescribing has not been established and needs to be assessed again at 12 to 24 months post intervention.

## Conclusion

The majority of antibiotic prescriptions in Australia are written in primary care but AMS is not well established in this setting. This study demonstrated the value of a simple AMS intervention comprising prescription auditing, feedback and an educational intervention, which together, led to highly significant improvements in antimicrobial prescribing.

This study contributes evidence that clinic-level AMS interventions improve antimicrobial prescribing and should be utilised on a larger scale. This intervention, was well received, highly effective and inexpensive. Future research to investigate the sustainability of these improvements is required to determine the ongoing benefit of auditing and educational intervention.

## Data Availability

The datasets used and/or analysed during the current study are not available due to participant privacy but are available from the corresponding author on reasonable request.

## Abbreviations

AMR: Antimicrobial Resistance
AMS: Antimicrobial Stewardship
GP: General Practitioner
MTOP: Medical termination of pregnancy
PBS: Pharmaceutical Benefits Scheme
REDcap: Research Electronic Data Capture
